# Connecting Species-Specific Extents of Genome Reduction in Mitochondria and Plastids

**DOI:** 10.1093/molbev/msae097

**Published:** 2024-05-17

**Authors:** Konstantinos Giannakis, Luke Richards, Kazeem A Dauda, Iain G Johnston

**Affiliations:** Department of Mathematics, University of Bergen, Bergen, Norway; School of Life Sciences, University of Warwick, Coventry, UK; Department of Mathematics, University of Bergen, Bergen, Norway; Department of Mathematics, University of Bergen, Bergen, Norway; Computational Biology Unit, University of Bergen, Bergen, Norway

**Keywords:** mitochondria, chloroplasts, organelle DNA, genome evolution, phylogenetic comparison

## Abstract

Mitochondria and plastids have both dramatically reduced their genomes since the endosymbiotic events that created them. The similarities and differences in the evolution of the two organelle genome types have been the target of discussion and investigation for decades. Ongoing work has suggested that similar mechanisms may modulate the reductive evolution of the two organelles in a given species, but quantitative data and statistical analyses exploring this picture remain limited outside of some specific cases like parasitism. Here, we use cross-eukaryote organelle genome data to explore evidence for coevolution of mitochondrial and plastid genome reduction. Controlling for differences between clades and pseudoreplication due to relatedness, we find that extents of mtDNA and ptDNA gene retention are related to each other across taxa, in a generally positive correlation that appears to differ quantitatively across eukaryotes, for example, between algal and nonalgal species. We find limited evidence for coevolution of specific mtDNA and ptDNA gene pairs, suggesting that the similarities between the two organelle types may be due mainly to independent responses to consistent evolutionary drivers.

## Introduction

Mitochondria (MT) and plastids (PT, the class of organelles to which chloroplasts belong) are essential compartments in eukaryotic cells. Originally independent organisms, both organelle classes arose from endosymbiotic capture and subsequent evolution (Sagan 1967; Keeling 2010; Zimorski et al. 2014; Roger et al. 2017). This evolution has involved the dramatic reduction of the organelle genomes (mtDNA and ptDNA, respectively; generally, organelle DNA [oDNA]) (Hjort et al. 2010; Keeling 2010; Roger et al. 2017). This reduction has proceeded to different extents in different taxa (Johnston and Williams 2016; Janouškovec et al. 2017; Giannakis et al. 2022). Some jakobid protists retain over 60 protein-coding genes in their mtDNA (Lang et al. 1997), while some parasitic organisms retain only 3 and others have completely lost mtDNA (Hjort et al. 2010; de Paula et al. 2012; Maciszewski and Karnkowska 2019). Some Rhodophyta contain around 200 protein-coding ptDNA genes (Janouškovec et al. 2013), while some parasitic plants contain only dozens (Naumann et al. 2016) and some reduced forms of plastid—like the apicoplasts found in Apicomplexans (McFadden 2011)—even fewer.

The diversity of oDNA gene profiles across eukaryotes raises, among others, two dual questions. First, what are the properties of a *gene* that make it more or less likely to be retained in a given species? And second, what are the properties of a *species* that make it more or less likely to retain a given gene? Numerous hypotheses for the first question have been proposed. It has been suggested that genes encoding hydrophobic products are preferentially retained in oDNA due to the difficulty of importing remotely encoded hydrophobic proteins into the organelle (von Heijne 1986; Björkholm et al. 2015), including via mistargeting of mitochondrial products expressed in the cytosol, which may result in gene products with mitochondrial presequences being delivered elsewhere—demonstrated across different genes (Björkholm et al. 2017). The colocation for redox regulation (CoRR) hypothesis proposes that retaining essential genes in organelles—where they can be expressed in response to local stimuli—supports local control of redox poise and organelle behavior rather than relying on slow and indirect nuclear transport (de Paula et al. 2012; Allen 2015; Allen and Martin 2016). Economic considerations of the energy required by different encoding locations also support organelle retention of some genes (Kelly 2021). A role for organelle genes as long-term redox sensors, identifying bioenergetically competent cells, has been proposed (Wright et al. 2009). Data-driven comparison of hypotheses using cross-eukaryotic genome data has provided support for the retention of genes encoding hydrophobic products and products that are physically central in their functional complexes (supporting CoRR-like control) (Johnston and Williams 2016; Giannakis et al. 2022). Pronounced convergence in oDNA ribosomal protein-coding gene content across species has been found in support of CoRR-like pressures on gene retention (Maier et al. 2013).

Some of these mechanisms—probably in combination (Johnston and Williams 2016; Giannakis et al. 2022)—may explain some of the gene-to-gene variability in retention. But what of the dual species-to-species variability in number of oDNA genes retained? It is well known that many parasites atrophy organelle genomes to very reduced or even absent oDNA, probably as a result of reduced energetic demand resulting from their exploitation of host metabolism (Hjort et al. 2010; Keeling 2010; Roger et al. 2017; Giannakis et al. 2022). Self-pollinating and clonal plant species typically transfer more oDNA genes to the nucleus; theoretical work has demonstrated that self-pollination accelerates such transfer when nuclear encoding is beneficial (Brandvain et al. 2007; Brandvain and Wade 2009). The coupled effects of nuclear genome reduction have been hypothesized as a reason for relative gene richness of red algal ptDNA (Qiu et al. 2017). The “limited transfer window” hypothesis places bounds on the time period during which a given taxa can transfer organelle genes (Barbrook et al. 2006).

Outside these specific cases, no general picture (if even applicable) has yet emerged. One candidate is the “mutational hazard hypothesis” (Lynch et al. 2006), which ties gene retention to taxon-specific oDNA mutation rates, suggesting that organisms with low rates of oDNA mutation (for example, plants) can support more organelle-encoded content without risking deleterious effects of this content becoming mutated. This picture has support from some observations and competition from others (Smith 2016a), with several instances where the hypothesized relationship between mutation rate and coding content runs in a different way to that predicted. Another candidate contribution to a cross-species picture is an interpretation of CoRR which attempts to bridge intercellular and organismal scales. Here, when organelles are required to rapidly adapt to changing environmental conditions experienced by their “host”, they are predicted to retain more oDNA genes, to allow individual local control of organelles (Johnston 2019). Hence, organisms experiencing highly variable bioenergetic and metabolic demands from their environment would retain more oDNA genes, and those experiencing lower and more stable demands would retain fewer. This picture is supported to some extent by mathematical modeling (García-Pascual et al. 2022) and comparative analysis of links between ecological traits and oDNA gene counts (Giannakis et al. 2023)—although we must point out that this picture does not and probably cannot explain the full diversity of oDNA retention profiles across eukaryotes. Although this theoretical picture predicts that similar factors shape—to some extent—mtDNA and ptDNA evolution, this prediction has to our knowledge yet to be tested with data. Here, while accepting that seeking too tight links between MT and PT evolution can be misleading (Smith and Keeling 2015), we set out to explore whether a quantifiable relationship exists between mtDNA and ptDNA gene counts across eukaryotes.

## Methods

### Data Gathering and Curation

We used the pipeline from (Giannakis et al. 2022) to collect cross-eukaryote data on protein-coding oDNA genes from the National Center for Biotechnology Information (NCBI)'s Organelle Genome database (O'Leary et al. 2016) and to curate and unify annotations. This pipeline involves resolving the inconsistent annotation of genes across eukaryotic records via two branches. First, an unsupervised all-against-all BLAST comparison (Camacho et al. 2009) of all oDNA coding sequence (CDS) records is performed, followed by iterative hierarchical clustering and relabeling based on the BLAST outputs, to give sets of genes that form a connected network in the space of BLAST comparison metrics (regardless of original annotation). Second, a supervised process of relabeling annotations based on a manually compiled dictionary of annotation protocols across taxa is performed. The relabeled annotations from these two approaches are then compared and any inconsistencies manually resolved.

NCBI's Common Taxonomy Tool (Federhen 2012) was used to estimate phylogenetic topology. The Encyclopedia of Life (Parr et al. 2014), Wikipedia (Wikipedia 2023), World Register of Marine Species (Ahyong et al. 2023), the USDA PLANTS Database (USDA and NRCS 2023), World Flora Online (WFO 2023), and AlgaeBase (Guiry and Guiry 2023) were used to collate ecological information on species.

### Statistical Analysis and Visualization

We used *phytools* (Revell 2012), *ape* (Paradis and Schliep 2019), and *phangorn* (Schliep 2011) for phylogenetic construction and analysis. For phylogenetic linear modelling (PLM), we used the *phylolm* package (Tung Ho and Ané 2014) for linear regression accounting for phylogenetic correlation with generalized estimating equations (Paradis and Claude 2002). We used *nlme* (DebRoy 2006) and *lme4* (Bates et al. 2015) for linear mixed models and *pheatmap* (Kolde 2019) for clustering and analysis. We used *Oncotree* (Szabo and Boucher 2008) for inference of progression. *igraph* (Csardi and Nepusz 2006) provided network analysis and visualization support. For visualization, we used *ggplot2* (Wickham 2011), *ggpubr* (Kassambara 2020), *ggrepel* (Slowikowski 2021), *ggraph* (Pedersen 2020), *ggtree* (Yu et al. 2017), *ggtreeExtra* (Xu et al. 2021), and *ggVennDiagram* (Gao et al. 2021).

## Results

### Profiles of Mitochondrial and Chloroplast Gene Count Across Eukaryotes

**Fig. 1.**
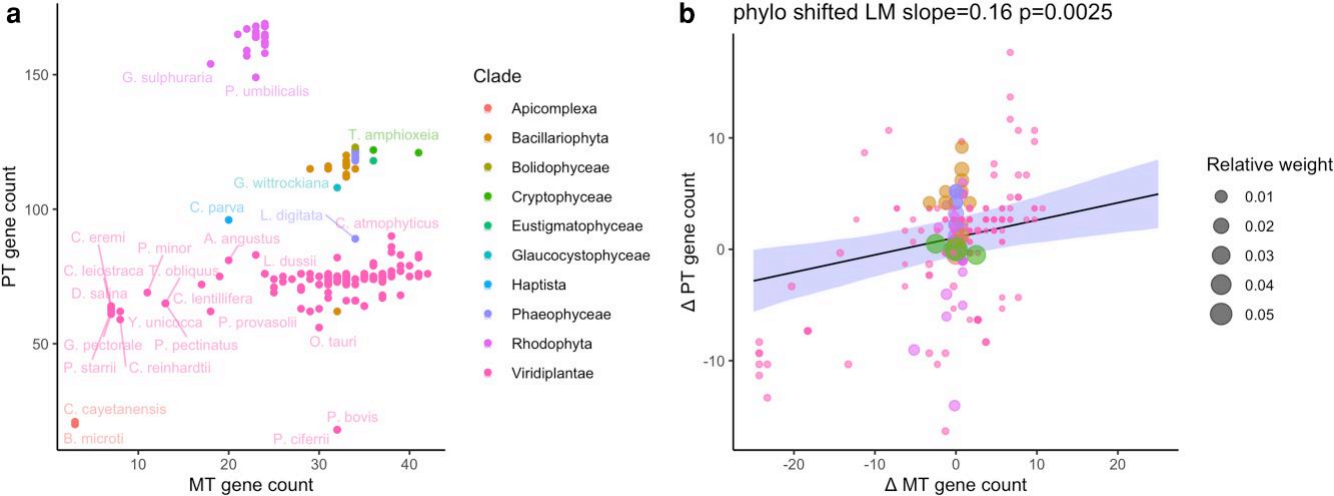
MT and PT gene counts across eukaryotes. a) Raw protein-coding oDNA gene counts across species in our dataset, labeled by basal eukaryotic clade. Some species are labeled for illustration. b) oDNA differences from the mean value for the species’ clade (ΔMT and ΔPT). Size of points in (b) is an illustration of their weighting in PLM: the inverse of the sum of their associated row in the variance–covariance matrix, reflecting a species’ relatedness to other members of the dataset. Sets of closely related species provide less independent information and so have a lower weighting. A PLM fit suggests a significant (*P* = 0.0025) relationship between ΔMT and ΔPT, also found in a linear mixed model for raw oDNA counts (supplementary fig. S1, Supplementary Material online).

We used the pipeline from (Giannakis et al. 2022) to collect cross-eukaryote data on protein-coding oDNA genes from NCBI's Organelle Genome database (O'Leary et al. 2016) and curate and unify annotations (see Methods). Following this pipeline, we found 205 unique species for which MT and PT gene counts were available. 147 were Viridiplantae (green plants and algae), 30 Rhodophyta (red algae), 11 Bacillariophyta (diatoms), 9 Phaeophyceae (brown algae), 2 Apicomplexa (typically parasitic protists), and representatives of other algal groups: 2 Cryptophyceae, and 1 each of Bolidophyceae, Eustigmatophyceae, Glaucocystophyceae, and Haptista. A simple plot of MT gene count versus PT gene count (Fig. 1a) shows considerable range and structure in the protein-coding gene count profiles. The Viridiplantae samples occupy a wide spread of MT counts at intermediate PT counts; Rhodophyta cluster at intermediate MT and very high PT counts; and other algae occupy a high MT/high PT region.

Several notable outliers were found from this data summary—all with lower PT gene counts than their closest relatives. These were *Laminaria digitata* (sublittoral brown alga), *Nitzschia alba* (coldwater diatom), and *Prototheca* spp. (parasitic algae). *N. alba* and *Prototheca* are both anomalous in the sense that they are apochlorotic members of their respective clades—having abandoned photosynthesis, their PT genomes have become reduced. *L. digitata* does not have this feature. Probing this case further, we found that although its chloroplast genome is described as containing 139 CDSs (Rana et al. 2019), its NCBI sequence NC_044689.1 only reports 107 CDSs. As its closest relatives in our dataset all have around 140 cpDNA CDSs, we marked this sequence as reflecting a possible missing data artifact, where not all genes are present in the reference sequence. The following analysis removes these outlier sequences, remembering that apochlorotic species depart from the trends we will report.

### Correlated Departures from Clade-Wide Gene Count Averages

To account for phylogenetic signal and pseudoreplication by relatedness (Freckleton et al. 2002; Paradis 2014; Maddison and FitzJohn 2015) in these observations, we changed variables to consider each species’ difference in oDNA gene count from its clade's average and performed PLM accounting for relatedness. Thus, the mean MT and PT gene counts across a clade were computed and then subtracted from each species’ individual counts to obtain a difference (ΔMT and ΔPT, respectively). A relationship between ΔMT and ΔPT, robust to phylogenetic correction, would suggest that those species with lower MT gene counts than their relatives also have lower PT gene counts than their relatives, and vice versa. In turn, this could suggest that similar pressures shape MT and PT gene counts at the species level.

A reasonably clear positive relationship from PLM (slope = 0.16, *P* = 0.0025) exists between ΔMT and ΔPT (Fig. 1b), although it must immediately be noted that a relatively small amount of the variance in one organelle gene count can be predicted by the other (*R*^2^ = 0.28). Supplementary fig. S1, Supplementary Material online, shows that, as expected, a linear mixed model with random effects assigned to clade shows a similar relationship (fixed slope = 0.39, *P* = 1.5 × 10^−15^).

To guard against artifacts from our chosen analysis method, we also analyzed the raw MT and PT gene counts using PLM (without shifting by clade mean) and the relationship between ΔMT and ΔPT using a linear model (without phylogenetic correction via PLM). All variants of this process showed a positive relationship between mtDNA and ptDNA gene counts: slope 0.56, *P* = 4.9 × 10^−5^ for PLM without clade mean shifting (equivalent to analyzing the points in Fig. 1a accounting for relatedness); and slope 0.38, *P* = 4.2 × 10^−15^ for a linear model after clade mean shifting (equivalent to equally weighting each point in Fig. 1b).

As Viridiplantae are the most sampled clade in our dataset, we also asked whether an MT-PT relationship was detectable within and without this clade. We used PLM to investigate the relationship between raw mtDNA and ptDNA gene counts in each case, finding significant relationships both within Viridiplantae (slope = 0.15, *P* = 0.0059) and without Viridiplantae (slope = 2.0, *P* = 6.5 × 10^−6^). Likewise, when Viridiplantae were removed from the linear mixed model, a connection was still detectable, albeit shifted (fixed slope = 0.98, *P* = 0.01); downsampling Viridiplantae to better balance the dataset (to 20 datapoints, becoming the second-most represented clade) recovers a fit resembling that from the original dataset (fixed slope = 0.41, *P* = 7.6 × 10^−7^).

### Ecological and Species-Specific Factors

**Fig. 2.**
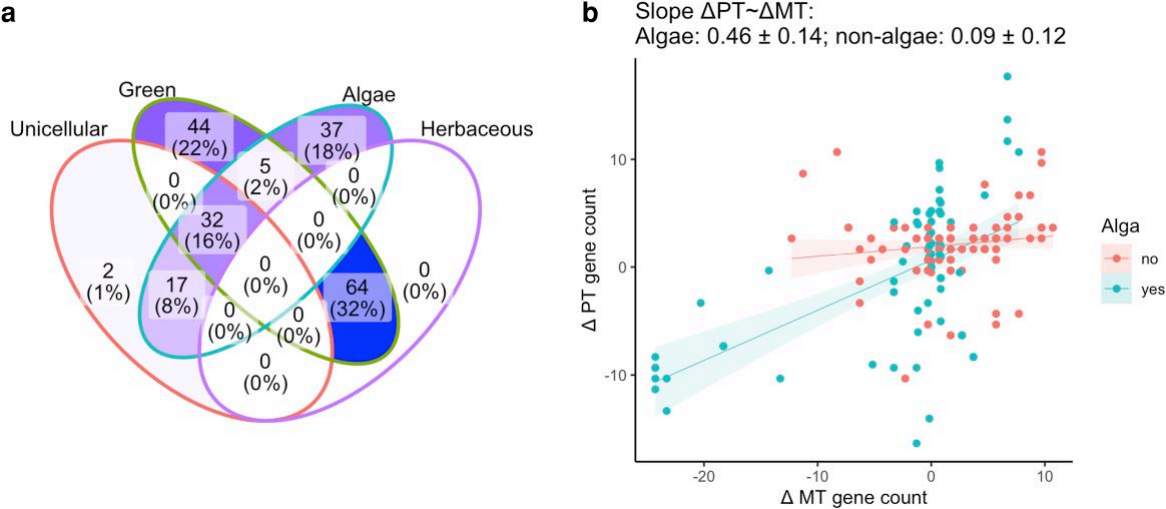
Species from different ecological categories in our dataset exploring the ΔMT–ΔPT relationship. a) Summary of different ecological classes of organism in the dataset. Numbers (and shading) give counts of species occupying each set of categories; percentages are the proportion of the total dataset. The two unicellular species that occupy no other sets are Apicomplexans. The herbaceous category is not applicable to algae or unicellular organisms. b) ΔMT–ΔPT for organisms labeled as algae and organisms not labeled as algae. Statistically robust differences in MT–PT behavior were not found for other arrangements of the features in (a).

Following work proposing ecological factors as shapers of oDNA evolution (García-Pascual et al. 2022; Giannakis et al. 2023), we asked whether ecological factors further separated substructure in the MT–PT space (Fig. 2). Following findings in Giannakis et al. (2023), aligned with the features that most broadly distinguished entries in our dataset, we tested for differences between organisms with different characteristics: algae versus nonalgae, unicellular versus multicellular organisms, green plants/algae versus others, herbaceous plants versus others, and annual versus perennial lifestyle. For clade-corrected oDNA counts (ΔMT and ΔPT), a linear mixed model with random slopes and gradients assigned to the algal category was slightly favored by Akaike information criterion (a statistic for selecting between models while guarding against model complexity) over the fixed linear model (1154.7 vs. 1157.5 for the fixed model). In other words, there is some statistical support for a picture where the relationship between ΔMT and ΔPT differs between algae and nonalgae (Fig. 2b). We found no statistically robust differences in oDNA behavior for other categorical differences between organisms.

### Gene-Level Relationships in mtDNA and ptDNA Evolution

We next asked whether there was evidence for evolutionary relationships between specific MT and PT genes—for example, is there a particular PT gene that is always lost when a particular MT gene is also lost? To this end, we used hierarchical clustering of the dataset to explore interdependence of PT–MT gene patterns (supplementary fig. S2, Supplementary Material online). We also used Oncotree, an algorithm for inferring relationships in progressive evolutionary processes (Szabo and Boucher 2008), to explore evidence for gene-specific dependencies in our data. Oncotree is designed for and applied to features in cancer progression but fundamentally deals with progressive evolutionary changes in binary traits.

**Fig. 3.**
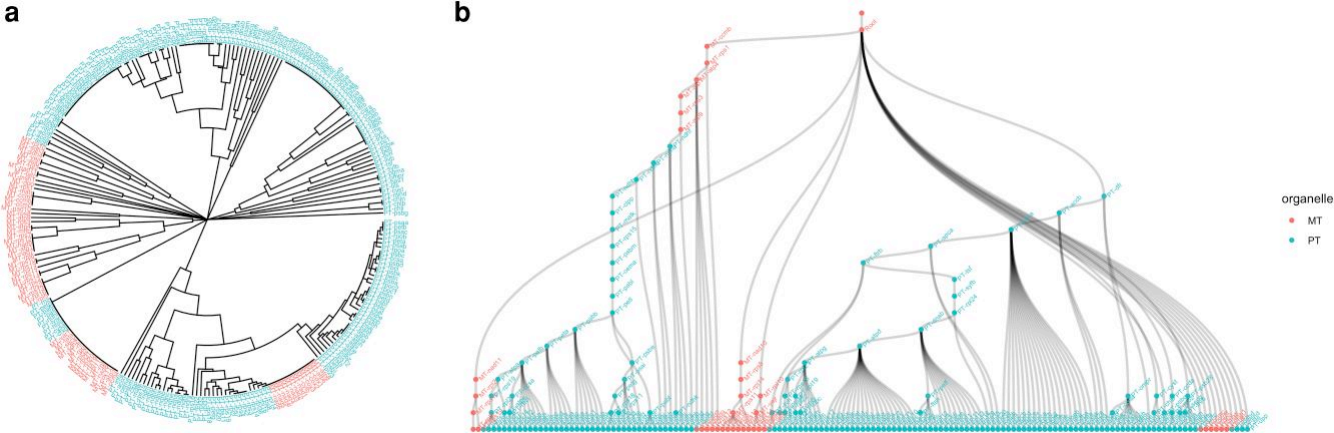
Limited evidence for gene–gene relationships across organelle types. a) Hierarchical clustering of gene retention profiles in our dataset (supplementary fig. S2, Supplementary Material online) produces a tree of relationships between genes, so that genes with similar retention profiles across species are the most “related”. Closest relatives in this tree are generally genes from the same compartment (MT or PT). b) Oncotree analysis (Szabo and Boucher 2008) of dependencies in MT–PT evolution. This analysis infers relationships between features in progressive processes (here, gene loss from oDNA), producing a tree where nodes higher on a branch are inferred to be lost before nodes lower on that branch. The tree here mainly shows gene–gene relationships within an organelle type. Some MT genes (top, left of center) precede a collection of PT genes, but the majority of genes cluster exclusively by organelle type.

We found no evidence of gene–gene correlations or conditional dependencies via hierarchical clustering of the data (Fig. 3a), with MT and PT genes clustering separately across the retention profiles in our dataset, rather than clusters of MT and PT genes together which could indicate subset-specific correlations. Oncotree suggested some consistent ordering behavior across organelles, where the loss of some MT genes (*ccmb, rps1, atp4, atp1, rps3, atp9*) is inferred to characteristically precede the loss of a collection of PT genes (Fig. 3b). This MT subset is not consistently among the most or least retained of organelle genes—their “retention indices” from (Giannakis et al. 2022) recording how many other genes are typically lost before them, take a range of intermediate values. The “dependent” PT subset overwhelmingly comprises highly retained genes (supplementary fig. S3, Supplementary Material online). Most other genes from both organelles clustered separately in this analysis too, suggesting only limited gene-specific relationships between organelles.

## Discussion

Our work suggests that, controlling for both phylogenetic signal and the relatedness of individuals, there is some support for a picture where MT and PT gene counts are connected. This does not appear to be manifest through pronounced cross-organelle interactions between specific genes (where, for example, the loss of MT gene X always results in the loss of PT gene Y). Rather, it appears that the drivers of reductive evolution apply comparably, but largely independently, to the two organelles. These drivers appear to be connected to some aspects of organismal lifestyle—algae and nonalgae, for example, display different MT–PT relationships.

This picture is compatible with recent results on both gene-specific (Johnston and Williams 2016; Giannakis et al. 2022) and species-specific (García-Pascual et al. 2022) drivers of oDNA genome evolution. Here, the same combination of features—hydrophobicity, guanine-cytosine (GC) content, and the centrality of a protein product in its functional complex—predicts a “retention index” describing how readily a gene is lost in either mtDNA or ptDNA. In parallel, the demands placed on organelle control by a species’ environment determine how many oDNA genes it retains. Broadly, those with higher retention indices are retained even by species in less varying environments; species in more varying environments retain genes with progressively lower retention indices. There are, of course, many more determinants of the oDNA retention profile of individual species—from the outcomes of stochastic evolutionary processes to population genetic considerations, and doubtless other mechanisms which the above picture does not consider. Correspondingly, the gene-specific models in (Johnston and Williams 2016; Giannakis et al. 2023) can predict just over half of the variance in gene retention indices (*R*^2^ = 0.51 to 0.60), and the relationship between MT and PT found here is certainly not clear-cut, with just over a quarter of variance in ΔPT statistically connected to ΔMT (*R*^2^ = 0.28; Fig. 1b). This figure is of course after removing the apochlorotic and potentially misannotated outliers in our data, the inclusion of which would lead to a still lower correlation.

In correcting for systematic differences in gene count between deep-branching clades (Janouškovec et al. 2017; Giannakis et al. 2022) and additionally for the phylogenetic relationship between samples, we have attempted to align with the picture in (Maddison and FitzJohn 2015), of both identifying independent subsets of data and then accounting “internally” for phylogenetic signal. However, how best to account for dependencies across phylogenetically embedded data is a topic of substantial discussion (Losos 2011; Uyeda et al. 2018); even well-principled comparative methods have shortcomings in the face of singular evolutionary events (Uyeda et al. 2018) and imperfect phylogenetic estimates (as we have here) and the distinction between pattern and process can challenge the application of these methods (Losos 2011)—discussed further in an oDNA context in Giannakis et al. (2023). In taking different combinations of clade-based and phylogenetic correction, and considering different subtrees of our data, we have attempted to be robust to particular choices of method. However, as more organelle genome data become available, testing alternative pictures like conditional dependencies between variables (Uyeda et al. 2018) will be an interesting line of inquiry.

The timescale of genome reduction must also be discussed. Janouškovec et al. (2017), for example, give a compelling picture where the majority of mtDNA gene loss occurred early in the history of most eukaryotic clades, with modern-day mtDNA being more (in some cases almost completely) static. Despite evidence of ongoing movement of genetic information from oDNA to the nucleus (Timmis et al. 2004)—for example, nuclear mitochondrial sequences (NUMTs) from mtDNA in humans (Wei et al. 2022) and very frequent ptDNA transfer in plants (Stegemann et al. 2003; Bock and Timmis 2008)—the gene profiles of many eukaryotic clades seem quite fixed. The two pictures can be reconciled by noting that although NUMTs and nuclear plastid sequences (NUPTs) can enter the nuclear genome, the functionalization of these fragments into a working gene and the subsequent removal of the organelle-encoded precursor presumably involve many more genetic and regulatory steps and can reasonably be considered a rare event in many species—as in the limited transfer window hypothesis mentioned previously (Barbrook et al. 2006). Overall, the patterns in gene content observed across modern oDNA samples were presumably largely established in the rather distant evolutionary past. Hence, the processes we study here are historical; and while modern-day oDNA profiles are clearly compatible with modern-day environments, it was not modern-day environments that originally shaped them.

We have only considered protein-coding gene counts across species—a very coarse-grained structural feature of oDNA. Tremendous diversity also exists in oDNA length and proportion of coding content, genetic structure (gene orderings and repeat regions), and physical structure (linear, branching, circular, and fragmented molecules) of oDNA across eukaryotes (Keeling 2010; Roge et al. 2017). Explanations for this diversity include the mutational hazard hypothesis (Lynch et al. 2006; Smith 2016a), effects of oDNA recombination in some lineages (Edwards et al. 2021), and recent work suggesting a functional link between gene order and the control of gene expression in Metazoa (Shtolz and Mishmar 2023); the interplay (or independence) of these mechanisms with the environmental influences we propose is an interesting target for future theoretical exploration.

Despite advances in sequencing protist oDNA, many clades are unrepresented in our dataset or only represented by a single species. Complete organelle records exist in the NCBI for over 9000 ptDNA sequences and over 2000 mtDNA sequences—but the large majorities of these are from green plants and animals, respectively (Smith 2016b). Organelle genome information from less represented clades—especially, for this study, photosynthetic protists—would be immensely valuable in verifying and refining these theories of organelle co-evolution. At the same time, such species play essential roles in marine and other ecosystems, and their photosynthetic and metabolic capacity and responses to environmental change are of profound importance on the warming and increasingly dynamic planet (Beardall and Raven 2004). More generally, oDNA features play an important role in the adaptability and evolvability of organisms in changing environments (Radzvilavicius and Johnston 2020, 2022), and environmental influences on photosynthesis and respiration are central to the biosphere's response to climate change (Dusenge et al. 2019). Understanding these essential genomes further, and the advantages and disadvantages of gene retention in different environments, is highly desirable in the study of diverse eukaryotic responses to a changing world.

## Supplementary Material

msae097_Supplementary_Data

## Data Availability

All data and code used in analysis and visualization are available at github.com/StochasticBiology/odna-coevolution/.
