# Supplementary figures and images for: Connecting Species-Specific Extents of Genome Reduction in Mitochondria and Plastids
Source: Mol Biol Evol. 2024 May 17;41(6):msae097. doi: 10.1093/molbev/msae097 (PMC11144018; doi:10.1093/molbev/msae097)

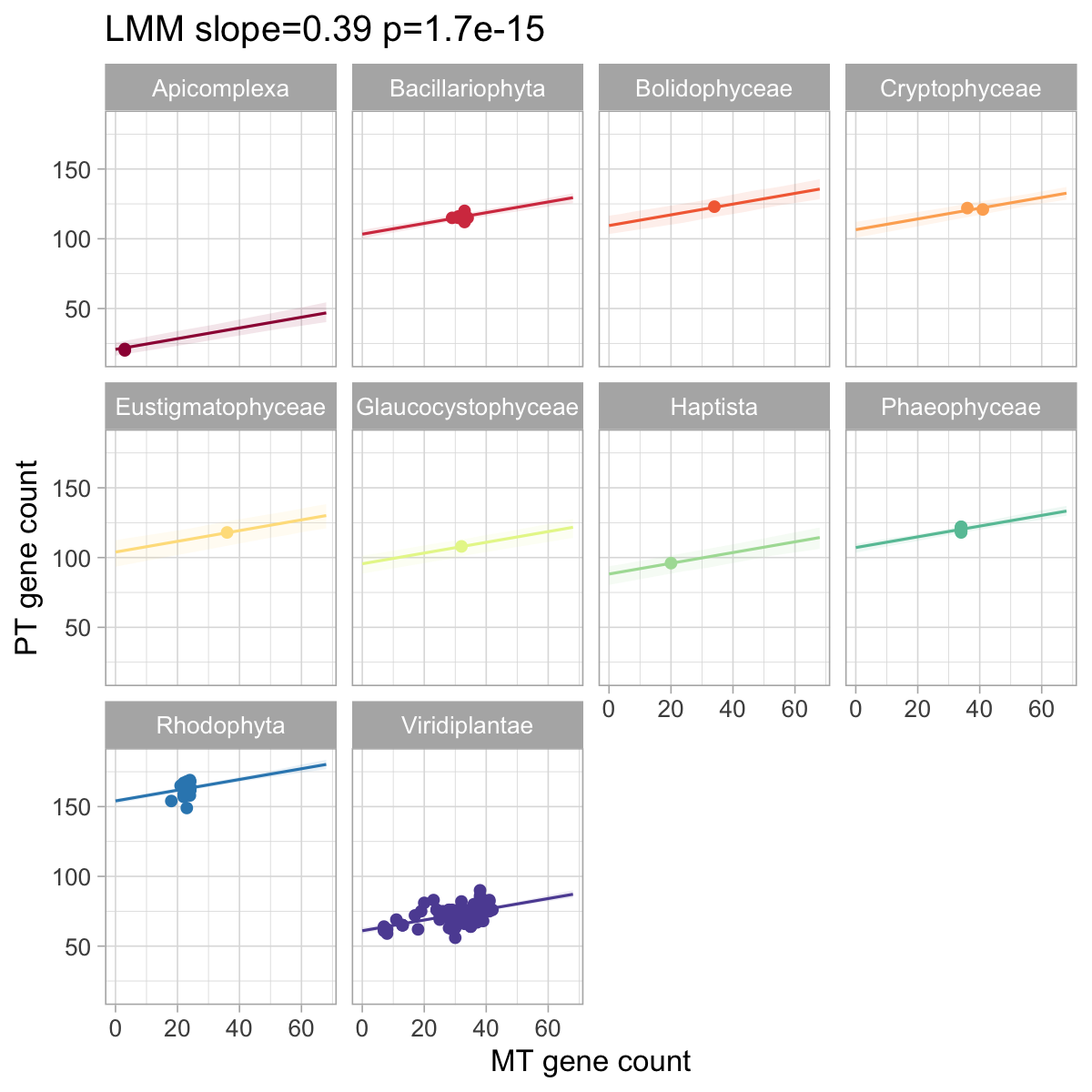

Supplement: msae097_Supplementary_Data [file msae097_supplementary_data.zip › fig-s1.tif]

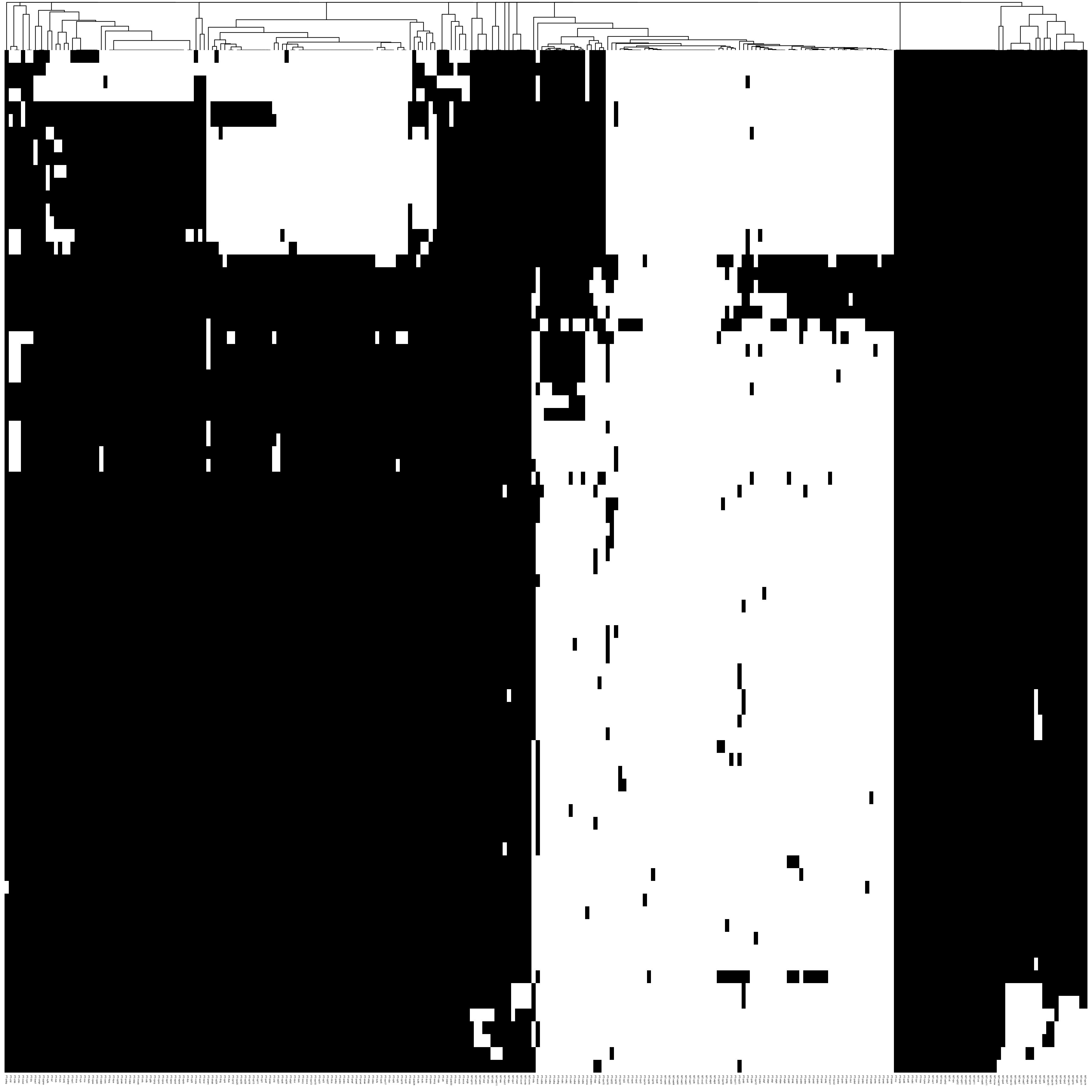

Supplement: msae097_Supplementary_Data [file msae097_supplementary_data.zip › fig-s2.tif]

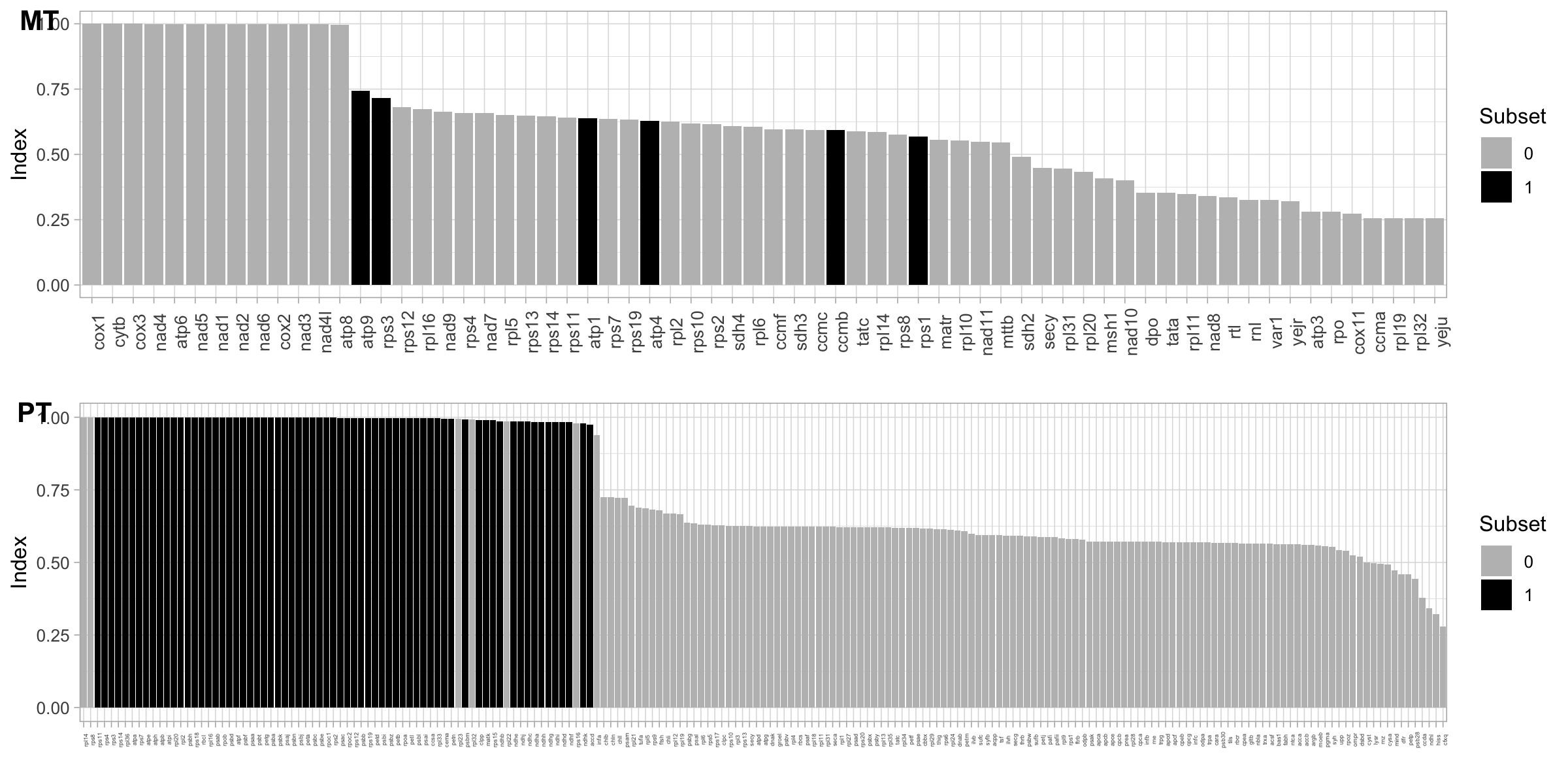

Supplement: msae097_Supplementary_Data [file msae097_supplementary_data.zip › fig-s3.tif]
